# Strengthening capacities among digital health leaders for the development and implementation of national digital health programs in Nigeria

**DOI:** 10.1186/s12919-020-00193-1

**Published:** 2020-07-23

**Authors:** Sunny Ibeneme, Nkiruka Ukor, Moses Ongom, Timothy Dasa, Derrick Muneene, Joseph Okeibunor

**Affiliations:** 1 Harvard T. H Chan School of Public Health, Boston, MA 02115 USA; 2 World Health Organization–Country Office, Abuja, Nigeria; 3 Federal Ministry of Health, Abuja, Nigeria; 4 World Health Organization–African Regional Office, Brazzaville, Congo

**Keywords:** Digital health, eHealth, Workshop proceedings, Nigeria

## Abstract

**Background:**

Discussions on the use of digital health to advance health have continued to gain traction over the past decades. This is important considering the rising penetration of mobile phones and other digital technologies and the opportunity to leverage those digital and electronic health methods and innovations to accelerate Universal Health Coverage (UHC) and the health Sustainable Development Goals (SDGs). In Nigeria, however, the full benefits of digital technologies to strengthen the health systems are yet to be fully harnessed due to critical challenges in the sector. These challenges include but not limited to weak health systems governance, weak infrastructural investments, inadequate resources, weak human resource capacity, high cost of scaling-up and coordination issues among others. Lack of systems thinking, and design have significant impact on coordination of efforts and has resulted in the fragmentation and non-interoperability among various applications. To discuss these challenges and propose the way forward for rapid sustainable, scalable and cost-effective deployment of digital health in Nigeria, a digital health capacity development workshop was held in Abuja and across the six geo-political zones of Nigeria from 20th – 30th November 2019. This paper documents key conclusions and achievements at the workshop.

**Methods:**

The workshop was organized around eleven modules and seven thematic areas which explored the Nigerian digital health governance and coordinating mechanisms in view of its status, leadership, financing and deployment for effective service delivery. It was attended by over 100 participants from varied background including Ministries of Health, Ministries of Communications and Digital Economy, International Organizations, Operators, Civil Society, Academia and Private Sector Experts.

**Results:**

The workshop resolved that while digital health technologies offer profound opportunities to strengthen Nigerian health systems for UHC and the health SDGs, there should be a move from donor-driven pilot projects to robust, sustainable, cost-effective and nationally owned projects. This will involve a people-centered approach that should be demand-driven and not supply-driven to avoid wasting time on ineffective interventions, duplication of efforts and wastage of scarce health resources. Government ownership and leadership was identified as critical for sustainable financing and effective scale up of Digital Health projects in Nigeria.

**Conclusions:**

The DH capacity development workshop was a good forum to deliberate important issues regarding sustainable and cost-effective DH solutions that could be scaled to strengthen health service delivery in Nigeria. Insightful ideas for scaling DH in Nigeria and other related settings emanated from the workshop, necessitating the need for a focused government commitment and leadership in institutionalizing digital health in Nigeria.

## Background

The World Health Organization (WHO) is committed to the highest level of healthcare services across the globe and is committed to facilitating member states to meeting global development goals including the 2030 Development Agenda– The Sustainable Development Goals (SDGs) and the Universal Health Coverage (UHC). The WHO Africa Region has continued to use innovative approaches to promote better health outcomes among member countries [1]. Among such innovations is the application of information and communication technology (ICT) services for health, referred to as Digital Health (DH). According to the WHO, DH includes all concepts and activities at the intersection of health and ICT, including mobile health (mHealth), Health Information Technology, Electronic Health Records (EHR), and telehealth [1, 2]. DH applications have been successfully deployed in countries of Africa to support numerous facets of health and had advanced the realization of UHC and health SDGs among African economies including Nigeria [2].

The World Health Organization Regional Office for Africa (WHOAFRO) and the International Telecommunication Union (ITU) in 2017 signed a cooperation agreement with the aim of building platforms to scale DH for UHC and health SDGs, build resilient health sector workforce to effectively use ICT infrastructure, and strengthen stakeholders’ partnership for sustainable DH adoption and implementation among African health systems. One of the pillars of the agreement is capacity building for the health workforce; others are interoperability, partnership, and medical devices for DH. Consequently, this presents an opportunity for DH to support a comprehensive and coherent approach to health and support integrated, people-centred health services [3].

Given the emerging importance of DH in support of UHC and the SDGs, and the recent World Health Assembly (WHA) resolution on DH, investments in DH capacity development for the health workforce is critical. DH systems and services cannot deliver better health outcomes without the necessary skills and competences from users, especially health personnel [4]. Capacity building for technical staff involved in the implementation of DH systems and services is critical to ensure rational use of technical tools, scale up and sustainability [5]. There is limited DH learning capacity among those able to implement DH systems at country-level or scalable levels [6]. Key workshop objective was to bring together DH leaders from Ministries of Health (MoH) and ICT who oversee the development and implementation of DH programs at the national and state levels, along with International Organizations, Non-Governmental Organizations, experts and donors to build participants’ capacity to lead DH initiatives.

The capacity building workshop was organized around eleven modules and seven thematic areas including– Understanding DH; National DH strategy development; DH interventions identification and requirements analysis; DH platform and applications design; Development, deployment, maintenance and scale up; Data use and analytics; and Monitoring and evaluation. Eleven modules were included for the training and include: Introduction to Digital Health; Digital Health Strategy, Governance and Regulations; Digital Health for Non Communicable Diseases (NCDs); Global Goods; Partnership models with telcos; Implementing Digital Health; Digital Health Architecture Design; Interoperability Framework; Monitoring, Evaluation and Learning; Digital Health Innovations, Big Data Analytics & Future Trends, and Global Health Security. Lectures were delivered using diverse approaches including face-to-face teaching sessions, high-level panel discussions, interactive sessions, and question and answer sessions to facilitate exchange of knowledge and ideas between participants and facilitators. Module facilitators were knowledge experts from WHO Country Office, Nigeria, Federal Ministry of Health (FMoH) Nigeria, Federal Ministry of Communications and Digital Economy Nigeria, Office of the Nigerian President Abuja, Nigeria, IT personnel from the Industry and development partners. Altogether, 182 participants across 36 states received capacity development through training.^1^ Two authors were assigned as Rapporteurs and documented proceedings from workshop discussions which was used to develop the workshop Report. This article summarizes the proceedings of the workshop and provides recommendations for effective establishment of national DH architecture in Nigeria.

## Understanding digital health

This module set the tone for DH discussions in the other eleven modules. It focused on understanding DH concepts and terminologies including the progress made so far in deploying DH applications in Nigeria. Keynote presentation highlighted opportunity for use of DH in advancing UHC and the health SDGs. The WHOAFRO provides support to 47 countries in the African Region, which is experiencing a rapid development of ICT, with 80.8% mobile penetration and 25.1% internet users’ penetration, against 99.70 and 47.10% at the world level [3, 7]. This proffers the opportunity for the use of DH to strengthen health systems for UHC and health SDGs among African economies.

Keynote presentation maintained that about 26 out of the 47 countries in the African Region had developed eHealth strategies including Nigeria. However, challenges remain in enabling countries to sustain DH services and the required human resources’ skills. These challenges include but not limited to non-interoperability among various systems, fragmentation of efforts, difficulty to monitor and to manage, high cost of scaling-up, uncoordinated investments, limited health systems impact and systems thinking. Keynote presentation reiterated improvements in the uptake of DH in the region, as evidenced in the last WHO eHealth survey, whose results were published in the WHOAFRO 2016 publication, called “Global Diffusion of eHealth” [2]. Subsequently, the Nigerian eHealth strategy was used as a model to highlight critical components including road maps for implementation.

During the question and answer session that followed keynote presentation, participants identified the need for capacity building on DH technologies and the documentation of DH achievements including the use of economic evaluation studies to build an investment model for DH. It was emphasized that individuals should be at the center of all DH initiatives to facilitate easy access to healthcare services removing geographical and time barriers. This module concluded by highlighting progresses made in the deployment of DH technologies in support of health service delivery in some parts of Nigeria despite persisting challenges. DH should not function in isolation but should be integrated into existing systems. Efforts should be made to ensure that DH projects are sustainable, scalable, cost-effective and adaptable in the context of any existing local systems.

## Digital health regulation, strategy and governance

Good leadership and robust governance mechanisms are central to achieving good health outcomes [4]. In view of this and the conclusions of module 1; module 2 focused on DH regulations and strategy including the relevance of governance in scaling DH in Nigeria. Important DH governance mechanisms were highlighted including the steering committees for strategic decision-making, the Technical Working Groups (TWGs) that provide expert advice and the Program Management Office that implement approved strategies and decisions. DH governance mechanism was further categorized into three including– Health ministry mechanism that drive DH and mobilize technical capacity and skills from other line-ministries, agencies, firms and organizations to deploy DH systems; Government-wide digital mechanism that provides significant ICT infrastructure and capacity; and DH agency mechanism that drive DH strategy and solution implementation. Digital health governance roles were enumerated to include program management, stakeholder engagement, strategic architecture, clinical safety, management and operations, monitoring and evaluation and policy oversight among others.

The Estonia government-wide digital model was used to explain basic concepts and how DH governance structures could be operationalized. Estonia model highlights the benefits of a DH approach based on a comprehensive eGovernment framework with basic structural elements including national electronic Identification, and system architecture designed for interoperability [4]. African countries that have thrived in this area were listed including Zambia, South Africa and Rwanda. Participants shared their experiences and success stories: One recurring theme was an integrated approach to DH governance for which state government played a central role in all DH narratives. The Ondo state resilient experience was used as case-study and key lessons include– Work with partners and vendors to make sure they use the same applications; collaborate with vendors to identify and fill gaps; concertedly come-up with strategy to address system challenges; coordinate support from government, policy-makers and other key stakeholders. The role of government cannot be over-emphasized in this course. Governments involvement and commitment are imperative in developing and implementing DH strategies, while providing robust leadership to coordinate the DH ecosystem which is often fragmented among health systems in Nigeria.

Keynote presenter maintained that DH strategy serves as a framework for planning, developing and coordinating various national DH initiatives. Elements that should be addressed in any strategy toolkit should be able to identify changes that system wants to strengthen as well as outcomes/outputs, action plans and governance mechanisms. Guidelines on how to develop national DH strategy were highlighted. Also outlined were overarching needs, desired activities, and outcomes; formulation of a DH investment plan to support national strategy; and establishment of a governance mechanism. Keynote presentation reiterated basic principles for responsible implementation of DH to include: Interoperability, sustainability, and multi-sectoral collaboration. The International Telecommunication Union (ITU)-WHO National eHealth strategy toolkit was mapped with the WHA71 resolutions on DH and overlapping areas identified including– Transfer of technology and knowledge, strengthening integrated people-centered health services, reuse and adaptations, interoperability, digital technology integration into existing health systems’ infrastructure, and implementations and scale up through DH strategies [5].

During the panel discussion session, keynote presentation reiterated the need for DH regulations and policies to adhere to international standards, and should be culturally adaptable, acceptable and accessible. Also stressed was the need to use systems thinking approach to engage stakeholders in DH governance and regulatory dialogues. The roadmap for strengthening DH regulations was addressed including systematic overview of the roadmap for health sector DH transformation. This gives details of how to move from having a national DH strategy to DH interventions identification and requirement analysis, up to the application design, development, deployment and Monitoring and Evaluation (M&E) of DH interventions.

This module concluded by recommending three basic concepts in the adoption of DH infrastructure including– DH governance mechanism, internal capacity building, and technical requirement gathering for systems. Emphasis was made on the central role effective governance and leadership play in scaling DH technologies in Nigeria. State governments should be committed to creating enabling environments for DH strategies to thrive, have a robust regulatory and financial mechanisms, and build strong partnerships to foster scalable, cost-effective and replicable DH initiatives that are adaptable to local contexts. States that does not have existing DH structures were encouraged to do so with promise of continuous support from FMoH and WHO Nigeria Country Office, Abuja.

## Digital health for non-communicable diseases

This module discussed the role of DH technologies in the control and prevention of Non-Communicable Diseases (NCDs) along with the challenges that impact DH implementation among developing economies. Conceptually, Africa is disproportionately affected by premature mortalities from NCDs along with other communicable diseases [7]. There is need to institutionalize infrastructure that guarantees long-term access to cost-effective interventions in the areas of prevention, diagnosis, treatment and rehabilitation. There is a growing evidence that behavioral risk factors for NCDs are rising among Africans– physical inactivity, unhealthy diets, tobacco use and excessive consumption of alcohol. Consequently, the introduction of best buys including alcohol and tobacco control interventions, increased vaccination and physical activity programs and timely screening for cancers are imperative in reversing the rising trend of NCDs in Africa [7].

The control and management of NCDs in Africa is fraught with myriad challenges including barriers to regular medical consultations, diagnostics, poor access to health promotion programs and pharmacotherapy especially among hard-to-reach populations. These challenges could be addressed by DH technologies [6]. The increasing mobile phone and internet penetration rates in Nigeria has opportunities to leverage DH technologies to prevent and control NCDs. Specific cases were cited including: “Be Mobile Be Healthy” which is a WHO-ITU initiative that uses mobile technology to improve the health outcomes for NCDs [8]. Important examples of this initiative include the mDiabetes of Senegal [9], mTobaccoCessation of New Zealand [10] and mCervical Cancer of Zambia [11]. Digital health is not monolithic but has multiple applications in solving health systems’ challenges. Keynote presentation demonstrated the use of DH tools as catalyst to optimize health interventions. Specific models were drawn from Reproductive and Maternal Child Health and NCDs. Different DH interventions address different health systems challenges and may intersect to solve health systems challenges to optimize health outcomes. This was operationalized using the DH intervention catalogue.

During the question and answer session that followed keynote presentation, participants maintained that capacity building at the State Ministry level to manage DH solutions was imperative; also, was the need to compute Return on Investment for DH and related economic evaluation results to justify investments in DH. Emphasis was made on the need for continuous technical support and guidance from FMoH and WHO. However, the need to involve the private sector including relevant ICT companies was addressed and strategies for engaging them discussed exhaustively. To ensure that there is in-country capacity, it was stressed that local ICT companies need to be attracted to provide DH solutions using robust business models that encourage private sector involvement.

This module concluded that DH plays a central role in achieving universal coverage for the prevention and control of NCDs in Africa. These roles include reminding patients on follow-up appointments, medication refills, and routine annual well-checks among others. Approaches that dissuade verticalization of NCDs programs were appraised, and the need to adopt system thinking approaches through collaborative efforts and strategies were emphasized.

## Global goods

This module discussed elements under DH global goods including: District Health Information Software 2 (DHIS2), Open Health Information Mediator (OpenHIM), Digital Health Atlas (DHA), Open Smart Register Platform (OpenSRP), Open Medical Record System (OpenMRS), Open Health Information Exchange (OpenHIE), Open Logistics Management Information System (OpenLIMS), Integrated Human Resource Information System (iHRIS), Open-source Laboratory Information System (OpenELIS), RapidPro, Open Deliver, and Telemedicine among others. Health is an information business [12]. As a result of the rising fragmentation and duplication of health systems and increasing proportion of information solutions being siloed, it becomes more difficult to share information across different systems, facilities and locations. This lack of interoperability hampers the effective use of information systems necessitating the need to move from architecture approach to interoperability approach scalable by the global goods [6].

Global goods comprise software, services and content. They enable systems to talk to each other using Open Standards, Open Data, Open Source and Open Innovation and have opportunities to improve health information sharing, processes of care and overall population health outcomes [13]. Keynote presentation highlighted prototype implementation guidelines for the global goods to include establishment of project steering committee; Identify system key-users; Define scope of the project; Identify funding sources; Measure baseline situation; Identify functional and technical requirements; Develop missing functionality; Have the results tested and validated by key-users iteratively; Setup mixed local network covering all functional entities of the facility. Others include installation of hardware with separate production, development and training Server environments.

Keynote presentation gave a high-level summary of the global goods: The DHIS2 was described as an open source web-based Health Management Information Systems (HMIS) software used for routine and non-routine aggregate data and patient-based data management. The OpenHIM was described as an open source with a middleware component that fosters interoperability between other global goods. It orchestrates and facilitates data flow between systems. The DHA was noted to be a global web platform to curate DH interventions and shared asset infrastructure investments, supporting governments, donors, technologists and implementers to map, monitor and foster DH investments to meet government strategic health goals. The OpenSRP was said to be an open-source DH platform that helps frontline health workers improve service delivery. It enables policymakers and program managers make informed decisions through direct access to robust data. The OpenMRS is a software platform with customized Medical Record System. It improves healthcare delivery in resource constrained settings by coordinating a global community that creates robust, scalable, user-driven, and open source medical record systems. The Kenyan eHospital powered by Bahmni, and the eSaude of Mozambique were cited as notable examples. The OpenHIE was described as a global mission-driven community of practice dedicated to improving the health of the underserved through open and collaborative networks, and the development and support of country-driven health information sharing architecture. The MomConnect of South Africa was cited as an exemplar use case [14]. The OpenLIMS was described as an open-source, cloud-based LMIS that manages health facility commodity supply chains. The LMIS initiative reduces the burden on healthcare workers, improves data accuracy, and fosters process timeliness for effective service delivery. Participants were worked through the Malawian OpenLMIS demo and the Sistema Electronico de Logistica de Vacinas of Mozambique.

Use-case that features iHRIS as part of the mHero communications platform was used to operationalize the iHRIS system. First deployed in Liberia during the Ebola crisis, mHero linked the iHRIS-based Health Workforce Registry with the DHIS2-based HMIS. The OpenELIS software is an open enterprise-level laboratory information system built on open source web-based technologies that has been tailored for low-and-middle income public health laboratories. It has ease of interoperability based on defined international standards and was typically used in the Cote d’Ivoire viral load dashboards. RapidPro was described as an open-source platform that allows anyone to design messaging interactions that can reach the most vulnerable populations. The mHero Ebola application of Liberia, the FamilyConnect of Nigeria and the mTrace child rights social messaging tools of Kenya were cited as notable use-cases. Open Deliver was described as a process for storing, creating, adapting, delivering and evaluating digital content. Some of the products under Open Deliver include ORB, OpiaMobile, Joomla, Moodle, MoodleMobile, Drupal, OPEN edX, OPEN edx-Client. Notable implementation examples include the OppiaMobile project of Ethiopia and the iDEA project of Nigeria [15].

Keynote presentation appraised the role Telemedicine has played in addressing challenges peculiar with the Nigerian health systems. It was identified that key health system challenges were how to de-isolate care professionals and improve healthcare service delivery in remote settings: This includes how to provide quality care in remote settings; how to recruit and retain healthcare professionals in the remote Nigerian communities; and how to keep up to date their knowledge and skills. To bridge this health system chasm, keynote presentation stated that the RAFT Network has done tremendous job in this regard by supporting healthcare professionals where they are most needed through E-Learning and Telemedicine platforms. The role Bogue (tele-expertise) software has done in this regard was appraised. The Bogou software, deployed in 2012 uses organized virtual community circles to collaborate remotely in the management of difficult clinical cases.

In the plenary session, basic implementation and sustainability challenges were highlighted and categorized into social and organizational; human resources; partnership, economic and financing; policy, legislation and proposal; and research, innovation and software developments factors. Others include uncoordinated investment and development, and reinvention of tools; inability to compare digital system functionality in standardized ways; and inadequate registry mechanisms that offer value to stakeholders. These resonated with state-specific challenges cited by participants including interoperability issues; cost, workflow disruption, privacy and data quality concerns, lack of technical support and incentives, complexity and limited computer skills among frontline personnel.

This module concluded by enumerating a prototype plan for implementing global goods which includes but not limited to– Establish a governance model and planning team including a project manager; identify what is needed and develop a plan; understand and use human-centered design; train and communicate plans; understand and embrace change management; make plans for maintenance and sustainability including clinical maintenance, technical maintenance and troubleshooting.

## Partnership with Telcos

The fifth module highlighted DH business models and frameworks for engaging Private-Public Partnerships (PPP) for scaling DH investments. Robust investments in the areas of finances, human resources and infrastructure are essential in advancing DH in Nigeria. According to the WHO global survey on eHealth, financial investments for DH varies for countries in Africa, with about 72% of the total funding provided by external donors [16]. This status quo aroused curiosity and concern to the issues of sustainability. Consequently, this module evaluated varied business models designed to engage business partners and foster sustainable financing of DH programs. Keynote presentation highlighted frameworks to dialogue and engage partners including agreements through Memorandum of Understanding. The critical roles of some key ICT and non-ICT partners in supporting DH financing in Nigeria was enumerated, with the need for governments’ commitment for partnership to further develop PPP emphasized [16]. This provides the opportunities for Nigerian governmental systems to jointly negotiate DH prices through national economic experts, thereby increasing their bargaining power while lowering the cost of DH in Nigeria.

During the question and answer session that followed keynote presentation, the need for multi-sectorial collaboration within the DH ecosystem was emphasized. The FMoH, WHO, European Union and other partners were encouraged not to relent in supporting local capacities for funding and sustainability of DH projects in Nigeria. Participants also called for improved ownership and commitment on the part of Nigerian government in the areas of sustainable financing of DH programs to avert project failures [14]. This module concluded that government play a central role in all PPP DH dialogues. Return on Investment for DH should not only be seen from the financial standpoints, but through the lens of health systems thinking with respect to number of lives saved, number of comorbidities averted and overall impacts on population health outcomes [17].

## Implementing digital health

Module 6 was split into three sub-sessions including: Implementing DH session, Governance and Strategy session, and Stakeholders session. In the first session, keynote presentation emphasized the need to assess the readiness of DH enabling environment before implementation. This includes identifying modalities for developing business requirements and identifying pain-points. DH readiness assessment tools were listed and include: The Health Information System Interoperability Maturity Toolkit, the DHA and the Global Digital Health Index. Basic DH development principles were appraised including: design with the user; understand the existing ecosystem; design for scale; build for sustainability; be data-driven; use open standards, open data, open source and open innovation; reuse and improve existing systems; address privacy and security issues; use a collaborative and concerted approach. The national DH implementation experience was shared highlighting strengths and weaknesses. Key strengths include but not limited to strong government commitment, robust stakeholder consultations and substantial infrastructural access. However, notable challenges described include– poor financing, dearth in skills transfer from partners to local frontline personnel, multiple silos implemented, inadequate regulation and donor chaos.

During the governance and strategy session, information systems were described to be a combination of people and technology. Keynote presentation maintained that IT projects should comprise a multidisciplinary team that can manage every aspect of development, deployment, operations, and governance for optimal result. The Ugandan and Kenyan DH strategy and governance model were appraised. While the Kenyan Health Policy 2014–2030 employs a participatory approach, the Ugandan model had TWGs that oversee country health informatics policies, legislation and regulation including health informatics professional societies and communities of practice.

During the stakeholder engagement session, keynote presentation described DH projects as not standalone projects. Thus, collaboration and coordination between the health sector, ICT sector and other stakeholders is important in sustaining DH interventions. Good governance remains a critical ingredient for scaling-up DH projects as well as intersectorialism among stakeholders involved in DH projects.

In the question and answer session that followed keynote presentation, participants raised issues related to the sustainability of DH projects in Nigeria. Issues on partnerships with the private sector and the government were addressed, including frameworks for engaging stakeholders especially for funding and sustainability. Others were issues on quality of service provisions and the governance mechanisms behind implementations and deployment protocols. Frameworks on ways of adapting digital technologies to be encompassing to accommodate dynamic health systems was summarized. The session concluded with a reminder that understanding existing systems is imperative and entails understanding the structural, cultural and political economy of communities before designing DH solutions adaptable to local contexts.

## Digital health architecture design

**Fig. 1:**
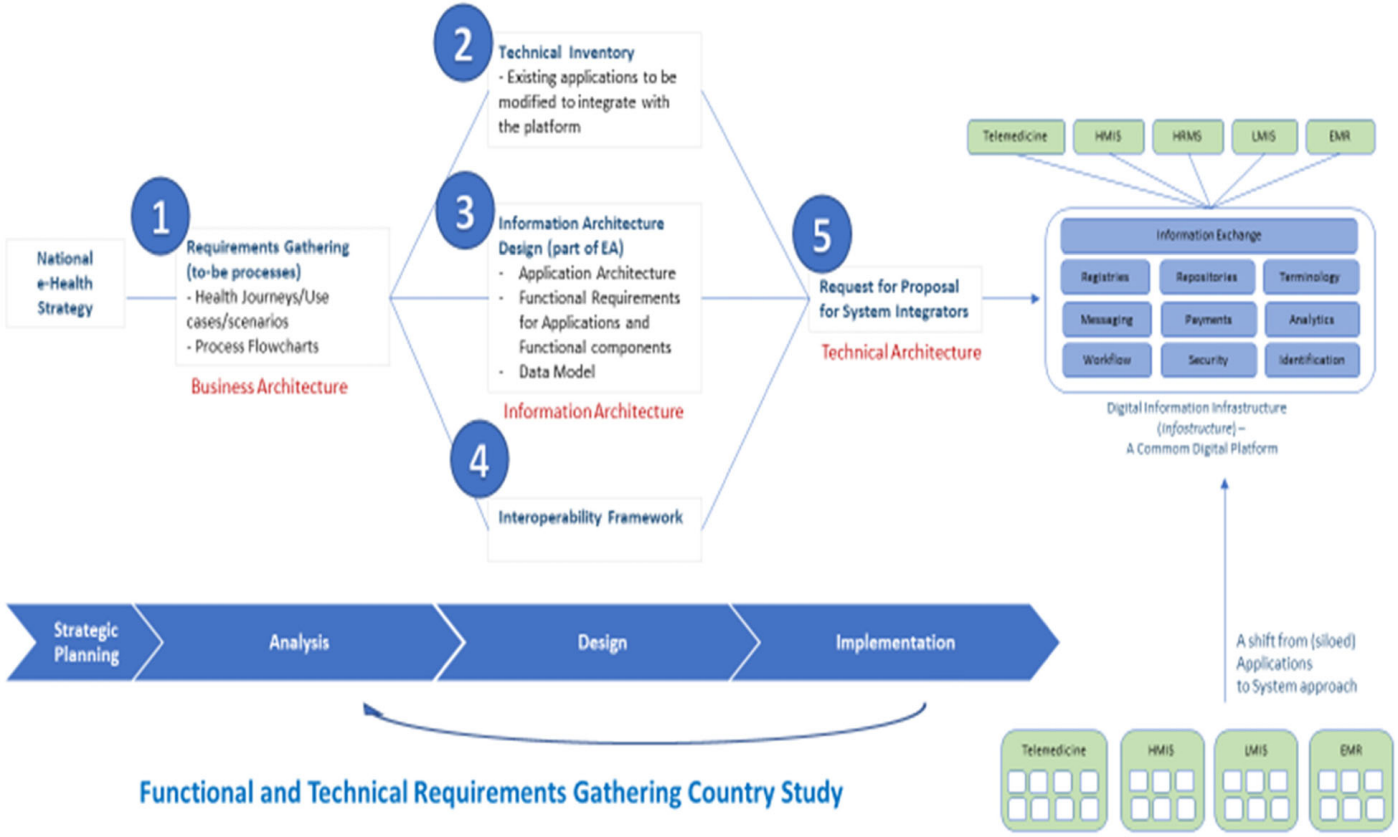
Establishing national digital health architecture and information infrastructure and system

Digital health architectural design was described as part of module seven including DH business architecture, information architecture and technical architecture. The Canadian Electronic Health Records blueprint was used to demonstrate enterprise architecture. Keynote presentation enumerated architecture requirements including: Business, Data, Application, and Technology architecture (Fig. 1). It was stressed that most skills and perspectives are often transferable from other sectors. However, transfer of business knowledge from other sectors to health is very minimal and limited. Thus, the need for a holistic approach to this course cannot be overemphasized.

The enterprise architecture breadth comprises enterprise vision and architecture definition, as well as related enterprise-level initiatives. The architecture-level components include segment vision and architecture definition, and segment-level initiatives. Keynote presentation worked participants through a new-user demo while using a pictogram to explain the link between national DH architecture and information infrastructure and systems (Fig. 1). The DH infostructure was described as a variety of purpose-specific digital software applications used by citizens, health providers, and administrators, which are connected to a technology platform that facilitates easy flow of clients’ health information in a secure and safe manner. It could also support a broad range of public health and care services, while gathering information from many sources that could help coordinate health systems’ planning and decision-making at all levels. Infostructure platform enables interoperability of DH applications through information flow and consolidates information from all sources in a shared health record (Fig. 1).

Keynote presentation maintained that business architecture involves translating strategy into digitally enabled processes to deliver business outcomes. Some of the basic inputs to business architecture are DH strategy, clinical guidelines, DH implementation handbook and DH platform handbook. Data architecture was described as the structure of an organization’s logical and physical data assets and data management resources. It outlines how data are generated, collected and used across applications including Digital Health Platforms (DHPs). DHP has an impact on application architecture. Applications in a platform-based environment need DHP common services. DHPs interact with all the DH applications used by patients and health facilities alike. Thus, DHP facilitates interoperability, enabling applications to provide needed information and participate in clinical and administrative workflow even though they are not directly integrated with each other.

In the plenary session that followed keynote presentation, participants stressed the need for the use of a Nigerian use-case to operationalize DH architecture designs. This could help horn down the issue and improve awareness among states in Nigeria. Issues related to investments in interoperability and the sustainability of DHP projects among Nigerian states were also raised for which purposeful government’s commitment was advocated. Government partnership with the private sector including frameworks for engaging stakeholders especially for funding and sustainability were further stressed. This module concluded by highlighting basic DH architecture considerations including: Considerations for the scope of DH for states which describes the extent of the infostructure architecture design based on available resources and implementation timelines.

## Interoperability, governance and framework

Keynote presentation maintained that the development of information systems in global health has often resulted in several systems residing in virtual silos. This lack of interoperability hampers the effective use of those information systems. Thus, the need to establish TWGs who make critical decisions regarding establishing interoperability standards cannot be over-emphasized. The TWGs set goals and standards including– standard terminologies, data exchange standards, and frameworks for documenting standards. For example, the OpenHIE is dedicated to establishing and promoting interoperability of Health Information Systems worldwide. It provides guidance on using freely available standards-based approaches and reference technologies, so developers can create connections between systems, and users can benefit from the interoperability. Interoperability according to keynote presentation was defined as the ability of systems, applications, and devices to communicate and share data with each other. This allows one system to speak the same language as another system, thereby enabling DH applications to talk to one another using standards which include but not limited to HL7, ICD-10, IHE DICOM, LOINC, WISN and SNOMED [15].

Keynote presentation also described standardized vocabulary as a fundamental enabler of semantic interoperability that allows data to maintain meaning regardless of who collected it, when or where it was collated, and what system that was used to capture it. Data standards are important to address inconsistent naming conventions, inconsistent definitions, and varying element values. It was further stressed that health information applications sit in a complex information eco-system. DH architecture facilitate interoperability and describes a solution for complex DH systems. The Eritrean National Health Management Information System (NHMIS) was used to operationalize interoperability frameworks. The Eritrean experience was an integrated, decentralized information system that includes data on vaccine stocks and enables the MoH to easily locate facilities with stock-out challenges.

During the question and answer session that followed keynote presentation, participants emphasized the need to make DH architecture, interoperability and implementation affordable across Nigeria. They highlighted diverse implementation challenges among various contexts including– Limited local capacity and governance in place to develop, endorse, and publish national data standards; Limited guidance on how to adapt international data standards to low-and-middle income country specific requirements; and limited guidance on best practices and how to leverage terminology services to exchange data in a way that drives context-specific health system goals.

This module concluded by reaching a consensus on the need for the creation and regulation of standards for the OpenHIE, including the standardization and harmonization of data dictionary for other global goods. It was also agreed for states to adopt international governance policies in directing interoperability, DH architecture and use of systems approach in all interoperability policy dialogues. Thus, fostering intersectoriality between MoH and International Standard Organizations that could ease adoption and implementation challenges.

## Digital health monitoring, evaluation and learning

This module justified the rationale behind conducting M&E for DH projects along with the bottlenecks related to M&E programs. Keynote presentation maintained that different stakeholders often have different M&E needs and are often curious to know if action plans are delivering anticipated results or not by appraising DH projects using basic M&E approaches including: Indicators– which defines identified indicators for DH projects; Measures– which defines baseline and target measures for indicators; Governance– which defines supporting governance and process structures.

Overall, DH M&E action plan includes inputs, processes, outputs, outcomes, and impacts. Inputs involves identifying DH output and outcome indicators, setting timeframes for M&E projects, having baseline and target measures for indicators and putting in place a robust governance mechanism for M&E activity. DH M&E outcome measure answers the question: What will be achieved or changed using DH tools? and how will the health system and services change because of the DH action plan. Output measures defines how the DH outcome could be achieved, and what the intended activities and deliverables could be. The implementation of M&E is fraught with challenges. However, the need to align implementation timeframe with M&E timeframe was stressed, and a demo used to demonstrate how to contextualize the design of M&E plans based on stages of implementation of the maturity toolkit.

The module concluded by emphasizing the need to choose the correct method to be aligned with the maturity toolkit; define the stage of maturity, stage of evaluation, and appropriate claims. Regarding the M&E governance; it was concluded that the governance model provides the structure by which collective parties involved in M&E align their roles in line with program charter.

## Digital health innovations, big Data Analytics & Future Trends

This module discussed the place of technology innovations within the DH ecosystem. The health sector is a complex adaptive system that relies on data for decision-making, advocacy and intervention mapping. This requires software applications, technology, data processes, metrics, incentives, skills, culture and sponsorship [6]. Keynote presentation used the Regional Action Data project to appraise health information generation and analytics within the digital environment. This include rationale on conducting information needs assessment, scholarly research gap identification, and framing of research questions among others.

Data analytics was described as an iterative process with feedback loops and require software applications. It denotes the extensive use of data including statistical analytics, explanatory and predictive models, and fact-based data interpretations to drive decisions and actions. In an era where Business Intelligence augmented by Machine Learning and Artificial Intelligence functions as are used for collecting, managing and reporting decision-oriented data. Thus, Business Intelligence have opportunities to facilitate analytical capabilities, processes and technologies in the health sector. Few models were cited where innovative technology have been used to optimize data management, training and implementation of healthcare service delivery.

In Ondo State, Nigeria; the state primary healthcare agency has used DH innovative technology approaches to improve health data quality; to train health workers remotely using video teleconferencing technologies, and to disseminate health-related information through the Short-Message-Services (SMS) technology. Zenysis technologies have developed platforms that integrate health data from routine information systems and surveys that coordinate mechanisms that facilitate health data interchange and comparisons used in predicting health events [4]. Babyl, a digital healthcare provider in Rwanda developed platforms for virtual medical consultation to reduce hospital waiting times. This system removes redundancy, improves system efficiency and performance. The Global Alliance for Vaccines and Immunization (GAVI) uses INFUSE (Innovation for Uptake, Scale-up and Equity in Immunization) an innovative digital technology to close the gaps in global immunization. GAVI also support other related projects that facilitates the distribution of lifesaving blood supplies using drones among rural health facilities of Rwanda [4].

During the plenary session that followed keynote presentation, panelist concluded that by its nature, developing an analytical capability is an iterative process, as managers gain better insights into the dynamics of their business over time by working with data and refining analytic models. Thus, quick and painless journey cannot be promised. It was further stressed that health sector data should be interoperable and accessible with less restrictions. There should be frameworks for translating and implementing data analytics and recommendations from academic research and related scholarly studies. Participants were updated on the Nigerian Health Observatories and the need for states to support FMoH and WHO Country Office in this regard by providing health statistics promptly. Regarding data exchange and access to data warehouse by researchers, a consensus was reached for data managers to always share de-identified data in-line with international data exchange standards as contained in the Health Information Exchange policy guidebook.

## Global Health security

Keynote presentation in the last module maintained that the WHOAFRO through the World Health Emergency (WHE) cluster gets involved in the early detection of acute public health events through event and indicator-based surveillance. They verify rumors and other related information regarding outbreaks and carry out objective assessment of outbreaks to guide responses and mitigation. At WHOAFRO, the AFROmobile Collect is an initiative by the Polio Eradication Program to encourage real-time mobile data collection across countries in Africa for health interventions with accountability as focus. It is built on the rudiments of Open Data Kit that allows seamless collection of field data for emergencies and routine surveillance expeditions through SMS and GPRS. WHOAFRO have developed systems including the Integrated Supportive Supervision, Electronic Surveillance, Auto Visual AFP Detection and Reporting (AVADAR) that integrate health data from diverse sources and facilitate easy surveillance, case-detection and reporting of Acute Flaccid Paralysis (AFP) poliomyelitis. These applications supported cholera interventions in Zambia, measles interventions in South Sudan and yellow fever coverage surveys in Nigeria.

In Democratic Republic of Congo, the KoBoCollect, a DH application used in health emergencies was successfully used to facilitate the timely sharing of information, rapid response, effective decision-making, and access to qualitative data. This corroborate the WHOAFRO EWARS in a bus project– an initiative of the WHOAFRO to strengthen early warning, alert and response in emergencies. It supports MoH and partners through the provision of technical support, training and field-based tools. This was an electronic mobile application that can be rapidly configured and deployed within 48 h of an emergency being declared. Thereby reducing the numbers of cases and deaths that occur during emergencies, through early detection, prompt validation and timely initiation of response [18].

Keynote presentation maintained that the use of DH technologies in outbreaks is fraught with several challenges and bottlenecks. Outbreaks and disasters are fluid and unpredictable [19]. Thus, inaccessibility in some areas contributes to weak surveillance performance, and may contribute to displacement of populations and an increased risk of spread of the epidemic. Other challenges listed were related to issues with consistency, accuracy and integrity of data; Lack of a database linkage system; Lack of standardized data collection tools; Lack of a centralized database system; and Lack of network infrastructure all of which could be addressed by DH technologies. During the plenary session that followed keynote presentation, the need to standardize system data especially those related data security and confidentiality protocols among global emergency systems were emphasized. National and local emergency protocols should adhere to international standards and guidelines. A consensus was reached on the need for multi-sectorial collaboration in containing emergencies to obviate effort fragmentation and duplication.

Participants called for emergencies coordinating bodies in Nigeria to harness the social media to this course, stressing with the increasing access to Facebook, WhatsApp, Twitter, and LinkedIn, emergency interventionists could tap from these resources to help inform public health outbreaks, surveillance and mitigation strategies. This module concluded that the earliest signs of an epidemic are digital, be it through search queries on Google, or through social media, or through patterns of movement, as can be approximated by movements of mobile phones, google search, and electronic messages and communications. Artificial Intelligence is invaluable in outbreak detection, as it proactively monitors the vast amounts of data that need to be examined to pick up the earliest possible signs of outbreaks [20].

## Conclusions

The DH capacity development workshop was a good forum to deliberate important issues regarding sustainable and cost-effective DH solutions that could be scaled to strengthen health service delivery in Nigeria. Insightful ideas for scaling DH in Nigeria and other related settings emanated from the workshop. First, while DH offers an opportunity for strengthening health systems towards the realization of UHC and health SDGs, the need to move from donor-driven pilot projects to more sustainable and longer-term nationally owned programs cannot be over-emphasized. Second, robust and resilient health systems should be a top priority of every DH initiative in Nigeria. Third, intersectorialism was encouraged among stakeholders in DH projects, as DH is no longer a standalone project. Thus, vendors are encouraged to partner with and engage local communities and governments to develop solutions that support communities especially the hard-to-reach populations to access quality and equitable health services. Fourth, vendors were encouraged to use Open Standards, Open Data, Open Source and Open Innovation. Using existing data and standards before inventing new ones and sharing data with the community is paramount in achieving success. Fifth, the need for government ownership, leadership, governance and PPPs in sustainable financing and effective scale up of DH in Nigeria was recommended. Sixth, deployment of DH applications should be demand-driven and not supply-driven, and efforts should be made to avoid duplications to ensure that specific public health challenges are addressed. Seventh, DH capacity building is imperative for health service delivery and human resource development. Eight, data security, protection and confidentiality remain an area for continuous improvement. The security of public health data is an important issue that needs to be addressed in Nigeria.

## Recommendations

We call on the Ministries in charge of health and ICT of all states in Nigeria and all stakeholders to ensure that the key recommendations of this meeting are implemented. Specifically, we call on the Nigerian Government to provide the required leadership for DH in the country. We also call on the private sector especially the private ICT Operators to support the Nigerian government to establish PPP for the scale-up of DH solutions in the country. Lastly, we call on Nigerian academic institutions to strengthen training on DH for health and ICT workers.

## Data Availability

Materials and information used in developing this manuscript were extracted from the final report of the digital health capacity development workshop and is available on request from the corresponding author.

## Abbreviations

AFP: Acute Flaccid Paralysis
DH: Digital Health
DHIS2: District Health Information Software 2
DHP: Digital Health Platforms
EHR: Electronic Health Records
FMoH: Federal Ministry of Health
GAVI: Global Alliance for Vaccines and Immunization
ICT: Information and Communication Technology
M&E: Monitoring and Evaluation
MoH: Ministry of Health
NCDs: Non-Communicable Diseases
PPP: Private-Public Partnerships
SDGs: Sustainable Development Goals
SMS: Short-Message-Services
TWG: Technical Working Group
UHC: Universal Health Coverage
WHA: World Health Assembly
WHO: World Health Organization
WHOAFRO: World Health Organization African Regional Office

## Footnotes

1 36 states include Abia, Adamawa, Akwa Ibom, Anambra, Bauchi, Bayelsa, Benue, Borno, Cross Rivers, Delta, Ebonyi, Edo, Ekiti, Enugu, Federal Capital Territory, Gombe, Imo, Jigawa, Kaduna, Kano, Katsina, Kebbi, Kogi, Kwara, Lagos, Nasarawa, Niger, Ogun, Ondo, Osun, Oyo, Plateau, Rivers, Sokoto, Taraba, Yobe, Zamfara State.
